# Medical Opinion and Movement

**Published:** 1911-06-17

**Authors:** 


### Medical Opinion and Movement.

### Pernicious Anaemia.

A remarkable case of pernicious anæmia is recorded by Dr. Walter, of Marbourg, in which a cure appears to have been effected by means of intra-muscular injections of blood from a woman suffering from splenic polycythæmia. The patient was a man aged fifty, showing all the typical signs of pernicious anæmia: rapidly progressing cachexia, extensive œdema, ascites, bilateral hydrothorax, and frequent and severe hæmorrhages of the nose and fundi oculi. The skin was extremely pale. The red corpuscles, numbering at first 2,500,000 per cubic millimetre, fell in the course of a few months to 700,000; the number of white corpuscles 4,100; and the hæmoglobin content 20 per cent. Microscopic examination showed intense poikylocytosis and nucleated red corpuscles, also megoblasts in small numbers. The patient was treated with iron, arsenic injections, intestinal antiseptics, etc., without any improvement. Intravenous injections of defibrinated blood gave rise to alarming symptoms of dyspnœa and irregularity of pulse, and had to be discontinued. Dr. Walter then had recourse to the treatment mentioned above. The blood from the polycythæmic patient was defibrinated and filtered through gauze and then injected, first 10 c.c. and later 20 c.c., into the muscles of the buttocks every five to eight days. After four injections the red corpuscles increased to 1,500,000, the white to 5,500, and the hæmoglobin content to 30 per cent. The dyspnœa also disappeared and the œdema diminished. After the ninth injection the red corpuscles amounted to 2,500,000 and the hæmoglobin to 40 per cent. The œdema completely disappeared and the general condition of the patient was excellent. After the eighteenth injection the red corpuscles were 6,600,000, leucocytes 6,500, and the hæmoglobin 90 per cent. While admitting the occurrence of marked remissions in this disease independently of any treatment, the author points out that the improvement in this case was so absolutely concurrent with the treatment that it is hardly possible to exclude a causative relation between the two. Moreover the improvement has persisted now for nine months.

### Elimination of Microbes.

An interesting series of researches has been carried out by Dr. Hess to determine the elimination of micro-organisms through the intestinal walls after introduction into the blood. An account of the work appears in the *Archives of Internal Medicine.* Tubercle bacilli injected into the veins were found in the stomach and intestines three hours later. Similarly, the Bacillus prodigiosus was isolated from the intestine one hour after injection. In order to exclude the possibility of access to the alimentary canal through the upper respiratory passage, the pylorus was previously ligatured in two cases, and in both positive results were obtained. With a view to exclude also elimination by the bile-duct this was first ligatured, and also the canal of Wissung in rabbits, and in the majority of cases the B. prodigiosus was found in the intestine soon after injection into the jugular vein. Similar results were obtained in dogs. In one dog a duodenal fistula was established with a double opening, the upper portion receiving the gastric contents, bile, and pancreatic juice, the lower being used to feed the animal. Two days later the two openings were closed and a culture of the B. prodigiosus injected into the jugular vein. Two hours later the animal was killed. Cultures from the small intestine showed the presence of the B. prodigiosus. Dr. Hess finds however that the bile constitutes the chief passage of elimination of microbes introduced into the blood stream, whereas the kidneys play a much less important rôle in this respect.

### Radio-dermatitis and Malignancy.

At a meeting of the Académie de Médecine, Dr. Pierre Marie reported that he had succeeded in obtaining the development of a malignant tumour in a white rat as a result of dermatitis produced by exposure to radium. He recalls the fact that he was similarly the first to show that malignant disease could be produced experimentally by exposure to the $x$-rays. An intense ulcerative dermatitis was first produced in the skin over the sacrum of a white rat aged five months by exposure to 70 H unfiltered radium rays in four séances. Imperfect cicatrisation took place in the course of a month. This was repeated four times. The fourth time an intense dermatitis resulted, although the dose was much weaker—only 2 H. This ulceration did not commence to cicatrise till six months later, and at the same time a neoplasm developed. The tumour grew rapidly. After extirpation it was found to present the structure of a sarcoma. Grafted on young rats it developed with the same morphological characters, and was transmitted in this way four times.

### The Advantages of Nephrostomy.

At a recent meeting of the Société de Chirurgie, M. Willems reported a case of vesical tuberculosis accompanied by great pain, in which he had performed a lumbar deviation of the urine. This operation was followed by disappearance of the pains, and a cure. Death did not occur until two years later. At the autopsy it was found that the vesical lesions had healed. This observer insists (1) on the efficacy of vesical exclusion; (2) on the advantages of nephrostomy, a procedure which is preferable to intestinal implantation of the ureters. When dealing with cachectic cases it is important to make the operation as short and as simple as possible, and, moreover, intestinal implantation means the risk of an ascending infection, whereas incontinence, the only annoyance, following nephrostomy can be easily overcome by the use of a good apparatus.

